# Supplementary material for: Characterization of neurobehavioral pattern in a zebrafish 1-methyl-4-phenyl-1,2,3,6-tetrahydropyridine (MPTP)-induced model: A 96-hour behavioral study
Source: PLoS One. 2022 Oct 3;17(10):e0274844. doi: 10.1371/journal.pone.0274844 (PMC9529090; doi:10.1371/journal.pone.0274844)
Supplement: Attachment — Submitted filename: Response to Reviewers.docx [file pone.0274844.s001.docx]

RESPONSE TO REVIEWERS’ COMMENTS

**RESPONSE TO REVIEWER #1:**

**Reviewer comment:** *The work presented by Khairiah Razali et al. aims at describing the impact of MPTP treatment in zebrafish. They analyzed the effect of the drug on different locomotor assays in zebrafish before and after 24h and 96 h. They suggested that the treatment induced locomotor deficits that may be relevant to Parkinson’s disease. This topic is important since there is a lack of in vivo preclinical model that are mimicking human pathology, thus pertaining the development of therapeutic strategies. Overall, the experiment are well conducted and the results are of importance.*

*However, I have some recommendations.*

**Response:** We appreciate the reviewer’s kind words. We revised the manuscript in response to the recommendations as described below.

**Reviewer comment:** *The authors claim that treatment with MPTP would induce visual deficit that could explain some of the locomotor behavior. In order to address this important question, I would suggest the authors to realize optokinetic response experiment to determine if MPTP treatment affects visual acuity.*

**Response:** We agree and have added a few sentences describing the optokinetic response (OKR) experiment for zebrafish. We assert, however, that the OKR experiment was not performed in our study. We can plan to assess OKR in our next experiment. (page 20, line 48-461).

**Reviewer comment:** *The observed phenotype with the turn angle, the meander and the angular velocity necessitate prosper discussion with relevant citations in order to strengthen their conclusion on the Parkinson’s disease phenotype.*

**Response:** As recommended by the reviewer, we have added further discussion on the observed phenotype and supported it with relevant citations (page 18-19, line 416-435).

**RESPONSE TO REVIEWER #2:**

**Reviewer comment:** *A good manuscript.*

*Title needs some rephrasing.*

*Discussion needs to address better some other toxicological and biochemical mechanism involved in this.*

*Results are decent and well worked*

*Introduction looks ok.*

*Methodology also fine, with no flaws detected.*

**Response:** We thank the reviewer for the kind words. We have revised the manuscript based on the comments as described below. Please have a look at the revised MS version.

**Reviewer comment:** *Title needs some rephrasing.*

**Response:** The old title “A Stable Five Days of Hypolocomotion and Abnormal Swimming Pattern in 1-Methyl-4-Phenyl-1,2,3,6-Tetrahydropyridine (MPTP)-Induced Zebrafish Model of Parkinson’s Disease” is changed to

***Characterization of Neurobehavioral Pattern in A Zebrafish 1-Methyl-4-Phenyl-1,2,3,6-Tetrahydropyridine (MPTP)-Induced Model: A 96-hour Behavioral Study.*** (Page 1, Line 1-2)

**Reviewer comment:** *Discussion needs to address better some other toxicological and biochemical mechanism involved in this.*

**Response:** Changes have been made throughout the Discussion section, with ultimate focus on specific behavioural changes and MPTP mechanism of action. However, since the study is limited to behavioural analysis, we refrain from overly discuss the molecular and biochemical aspects of the behaviour changes to avoid misinterpretation.

**RESPONSE TO REVIEWER #3:**

**Reviewer comment:** *In the submitted MS Razali and coworkers report the behavioral change by a single dose of MPTP intraperitoneally administered to the adult zebrafish 1 and 4 days after injection. This is a methodologically sound and thorough study and the novel insight is basically the stability of behavioral changes between these time points, except a loss of increased freezing bouts at 4 days (see also specific point to page 12 below). This finding might be important for the use of the MPTP-model in zebrafish in the context of neuroprotective studies considering the well-known reversibility of the neurodegeneration induced by MPTP in this species.*

**Response:** We appreciate the reviewer’s positive feedback. We amended the manuscript based on the specific points as described below.

**Reviewer comment:** *Due to the focus of this rather methodologically circumscribed study, all discussions of mechanistic aspects of MPTP in the zebrafish are redundant (page 18, line 417 to page 20, line 458), which means that the discussion should be shortened considerably and shifted to a more thorough comparison of the specific behavioral findings to that reported in the literature (e.g. comparison to the study of Anichtchik OV et al., J. Neurochem. 2004).*

**Response:** We agree with the Reviewer. Changes have been made throughout the Discussion section. We have added a more thorough discussion focusing on the specific behavioural changes, including comparison with the suggested reference. (P17-L398-411)

**Reviewer comment:** *Page 3, line 54: Who says that cholinergic neurons are lost in the substantia nigra in PD?*

**Response:** The sentence has been corrected and revised (page 3, line 56).

**Reviewer comment:** *Page 3, line 55: A review on the promise Zebrafish in PD is not an appropriate review on the basic characteristics of the pathology of PD.*

**Response:** The reference for this sentence (Razali et al., 2021) has been changed to more appropriate references (DeMaagd et al., 2015 and Kouli et al., 2018) (page 3, line 57).

**Reviewer comment:** *Page 8, line 176-182 or page 9, line 196-202: Please specify the geometry of the IN and OUT zone within the recording tank.*

**Response:** We specified the geometry and dimension of the zones (page 8, line 180-183).

**Reviewer comment:** *Page 9, line 196-202: Please specify the definition of bouts in the context of freezing behavior.*

**Response:** We added a sentence defining the freezing bouts (page 9, line 206-207).

**Reviewer comment:** *Page 9, line 196-202: Please specify the definition of frequency in the IN zone in the context of swimming pattern.*

**Response:** We added a sentence defining the frequency and time spent in the IN zone (page 9, line 207-209).

**Reviewer comment:** *Page 12, line 262-264, page 12, line 273-275 (legend to Fig.5): Is there no statistical significance between 96h and 24h in Fig.5b for MPTP-injected fish?*

**Response:** Indeed, there is statistical significance of freezing bouts between 24h and 96h. But since we performed the analysis by comparing each time point with 0h, we did not indicate the significance between 24h and 96h. We did, however, added a sentence regarding the difference in the Discussion section (page 17, line 391-394).

**Reviewer comment:** *Page 13, line 287 (legend to Fig.6): Please specify for frequency in the IN zone during which time the numbers are counted (similarly to the way the number of freezing bouts are described in the legend to Fig.5b).*

**Response:** We revised the sentence accordingly (page 13, line 298-299).

**Reviewer comment:** *Page 18, line 422/423: The wording suggests that reference 36 (Miskitsh & Chacko, 2014) is reporting data about MPTP, which is not the case*

**Response:** We have amended and cited a more suitable reference (Brady et al., 2005).

**Reviewer comment:** *Page 18, line 424 to page 19, line 429: It is unclear why just reference 37 (Risiglione et al., 2022) is especially relevant for the degenerating effect of MPTP on dopaminergic neurons, a neurotoxic mechanism elucidated by studies decades earlier.*

**Response:** We added more references including those from earlier studies (page 21, line 481-488).

**Reviewer comment:** *Page 19, line 440-441: Reference 38 (Zinger et al., 2011), a review about neuroinflammation and kynurenine in PD, is not an appropriate reference for the indirect pathway in basal ganglia, first described decades earlier.*

**Response:** We have amended this paragraph.

**Reviewer comment:** *Page 19, line 450-452, page 20, line 454-456: Reference 41 (Huang et al. Parkinson’s disease 2017) is not a specific rodent study, but a review about changes in the MPTP mouse model.*

**Response:** We have revised and changed the old reference (Huang et al. 2017) to a more relevant reference (Alam et al., 2016) (page 21, line 490-495).

**Reviewer comment:** *Page 23-26, References: Please give more complete information for cited books.*

**Response:** We updated the citations for books (manuscript: page 25-29).

**RESPONSE TO REVIEWER #4:**

**Reviewer comment:** *This study investigates the neurobehavioral toxicity of a single i.p. injection of MPTP, a model chemical for Parkinson's disease, in adult zebrafish. The main merit of this study is a complete characterization of behavioral endpoints at 0, 24h, and 96h post MPTP injection. The behavioral analysis was rather comprehensive and in great details.*

**Response:** We thank the reviewer’s kind sentiments. We revised the manuscript based on the comments as described below.

**Reviewer comment:** *Both male and female adult fish were used in this study, but no mention about whether there was any sex difference in all these behavioral parameters.*

**Response:** We take note on the reviewer’s comment. However, since the main objective of this study was to measure the behaviour between MPTP-treated and untreated zebrafish, we did not compare between male and female fish as determining the sex differences require separate between-subjects statistical analyses on each parameter. We did, though, realize the significance of comparing male and female behaviour and have included this as a suggestion for future work (page 23, line 530-532).

**Reviewer comment:** Lack of histopathological examination of the MPTP-treated brain to confirm any DA neuron loss, though authors acknowledged this in their Discussion.

**Response:** We agree with the reviewer that the findings from this study is limited to the swimming behaviour only and lack molecular or histopathological examination. Nonetheless, we supported our deduction with published studies that are in agreement with our findings, to support our conclusion.

**Reviewer comment:** *A minor comment is that there are a few recent MPTP studies using the zebrafish were not cited and incorporated in the Discussion, for example: https://pubs.acs.org/doi/abs/10.1021/acschemneuro.2c00089*

[*https://www.sciencedirect.com/science/article/abs/pii/S0161813X22000997?via%3Dihub*](https://www.sciencedirect.com/science/article/abs/pii/S0161813X22000997?via%3Dihub)

**Response:** Changes have been made throughout the Discussion section, and we have included the reference suggested by the Reviewer.
